# Delayed Demyelinating Disease of the Central Nervous System Following Low-Voltage Alternating Current Electrical Injury: A Case Report and Review of the Literature

**DOI:** 10.7759/cureus.43951

**Published:** 2023-08-22

**Authors:** Valentin Morosanu, Rodica Balasa, Sergiu Morosanu, Beáta Baróti, Iulian Roman-Filip

**Affiliations:** 1 Neurology, Emergency County Hospital Targu Mures, Targu Mures, ROU; 2 Neurology, University of Medicine, Pharmacy, Science and Technology of Targu Mures, Targu Mures, ROU; 3 Cardiology, Targu Mures Institute for Cardiovascular Diseases and Heart Transplantation, Targu Mures, ROU; 4 Radiology, Emergency County Hospital Targu Mures, Targu Mures, ROU

**Keywords:** neuropathology, demyelination, neurological complications, alternating current, electrocution

## Abstract

Electrical injuries are relatively common types of mechanical trauma associated with significant morbidity and mortality. These injuries occur most commonly in adult men and account for approximately 3-7% of admissions to burn units. The type and amount of current, voltage, tissue resistance, and duration of current flow all influence the extent of injury and the patient outcome. A broad spectrum of central nervous system (CNS) and peripheral nervous systems (PNS) disorders caused by electrocution have been described in the literature. Here, we present a rare case of a 45-year-old man, electrocuted with a 240 V low-voltage alternating current (AC), four years prior to presentation, who has been admitted to our neurology clinic with a positive Lhermitte sign, paraparesis, proximal muscle pain, and distal paresthesia of the lower limbs, symptoms that had appeared one year after the electrocution. Magnetic resonance imaging (MRI) of the brain and spinal cord revealed multiple demyelinating lesions involving pons, juxtacortical and periventricular regions of the brain, and cervical and upper thoracic spinal cord. Given that other etiologies of demyelinating diseases of the CNS were excluded, we have interpreted this case and all accompanying pathologic findings as a consequence of electrical injury. Although the general epidemiologic reports regarding age, sex, type of current, circumstances, and site of electrocution correspond to the data of our reported case, this patient presents a delayed, rare neurologic complication with a nonspecific MRI pattern that we did not find in the literature. These patients should be carefully monitored not only during the acute phase but also over a longer period, because, as reported in this case, neurological complications may occur later after electrocution.

## Introduction

Electrical injuries represent an emerging type of mechanical trauma, usually produced accidentally by lightning and low-voltage (<1,000 V) and high-voltage (≥1000 V) currents, and account for 3-7% of admissions to burn units. Each year in Europe, there are about 16,000 people injured and 540 killed by electrocution, while in the USA, there are about 1,000 electrocution-related deaths annually and 40% of them are due to high-voltage electrical injuries [1,2]. Electrical trauma occurred most frequently in adult males, aged between 20 and 50 years, and in industrial workers and householders at home. Adult men are more exposed to this type of injury apparently because of additional interest in electrical appliances and engaging in high-risk professions [3,4].

Electrical currents can be classified as alternating (reverse conduction of electrons) and direct (flow of electrons in one direction). Common paths of electrical energy transmission are hand to hand, hand to foot, and head to foot, the entry point being the source and exit point - the ground [3,5]. The type and amount of current, voltage, tissue resistance, and duration of the current flow all influence the extent of injury and the patient outcome.

Lower-resistance tissues (nerves, blood vessels, and muscles) serve as good conductors for electrical current due to their high water and electrolyte levels, so central nervous system (CNS) and peripheral nervous systems (PNS) complications are well recognized [4].

The proposed pathophysiological mechanisms of both low- and high-voltage electrocution-induced CNS lesions are the following: thermal or direct mechanical damage of the neurons with secondary neuronal loss, microglial activation with blood-brain barrier dysfunction and neuroinflammation, neuroexcitotoxicity due to glutamatergic hyperstimulation, electroconformational denaturation of proteins and DNA, oxidative stress activation and neuronal ischemic changes due to vascular spasm, and endothelial inflammation. Electroporation is considered an important factor in neuronal alteration after electrocution as well, causing an immune-mediated demyelination following perforations in the phospholipid bilayer and myelin antigen structural damage [6-9].

It is a difficult task to rule out all the etiologies of some of these consequences and to quantify the actual impact of the electrocution on developing these conditions. The aim of this article is to report a rare case of late-onset demyelinating disease of the CNS after low-voltage alternating current (AC) electrical injury and to review the literature related to this topic.

## Case presentation

A 45-year-old man with no remarkable medical history was referred to our neurology clinic in 2019, complaining of lower limb weakness associated with proximal muscles pain, distal paresthesia, and an electric shock-like sensation that extends down the spine upon flexion of the neck.

The patient had been developing a progressive paraparesis for three years before the presentation to a regional hospital (2016), where a magnetic resonance imaging (MRI) of the brain and spinal cord had been performed. Scan sequences had shown multiple T2 hyperintense lesions of the cervical spinal cord, brainstem, and periventricular white matter, without gadolinium enhancement. No other specific paraclinical investigations than the usual ones had been performed at that time to elucidate the diagnosis. Intravenous corticosteroids had been administered for seven days with a slight improvement in symptoms.

It is important to note that four years prior to the presentation to our clinic (2015), he had suffered a 240 V AC electrical injury while operating a welding machine at home in a snowy weather, without any protective equipment. It was presumed that the electric current had entered the body through his right hand and caused continuous muscle contraction of the forearm flexors and extensors for about 15-20 seconds. He presented at that time to the emergency department, where the first degree burns of the right hand were noted, medical care was provided, and the patient was discharged home in good condition with no chief complaints, arrhythmias, or rhabdomyolysis.

At the time of presentation to our neurology clinic, we had noted the scar on the patient’s right hand, mild facial hyperemia, no signs of skin spots or moles suggestive for melanoma, and normal vital signs. Neurological examination revealed normal mental status, absent meningeal signs, intact cranial nerves, a positive Lhermitte sign, motor examination with the Medical Research Council (MRC) grading being 4/5 MRC for both lower extremity muscles, exaggerated deep tendon reflexes and bilateral Babinski sign, distal paresthesia of the lower limbs, and no bladder or bowel dysfunction.

The differential diagnosis of those small, multiple, symmetrical lesions in the brain and spinal cord included multiple sclerosis, neuromyelitis optica and myelin oligodendrocyte glycoprotein spectrum disorders, acute demyelinating encephalomyelitis, CNS vasculitis, sarcoidosis, viral infections, Lyme disease, HIV-related CNS diseases, metastatic disease, and autoimmune diseases, such as systemic lupus erythematosus, Sjogren syndrome, and Behcet disease. Therefore, the proper investigations to rule out all these pathologies were performed.

Complete blood count and serum chemistry had shown only a mildly elevated erythrocyte sedimentation rate (16 mm/h). Workup for infectious profile included HIV antibodies and Venereal Disease Research Laboratory (VDRL) test, *Borrelia*, *Toxoplasma gondii*, herpes simplex virus, cytomegalovirus, Epstein-Barr virus immunoglobulin G (IgG) and IgM antibodies, hepatitis B surface antigen (HBsAg), and hepatitis C virus (HCV) antibodies (all negative). Workup for autoimmune disease profile included antinuclear antibodies, antineutrophil cytoplasmic antibodies (cANCA and pANCA), anti-phospholipid antibody, and angiotensin-converting enzyme (all negative). Workup for paraprotein profile included cryoglobulins, serum immune fixation electrophoresis, peripheral blood smear, and serum vitamin B12 level (within normal values). Moreover, anti-acetylcholine receptor, anti-aquaporin 4, and anti-myelin oligodendrocyte glycoprotein (MOG) antibodies were negative. We had also performed a lumbar puncture, with a normal cerebrospinal fluid (CSF) analysis and negative oligoclonal bands.

**Figure 1:**
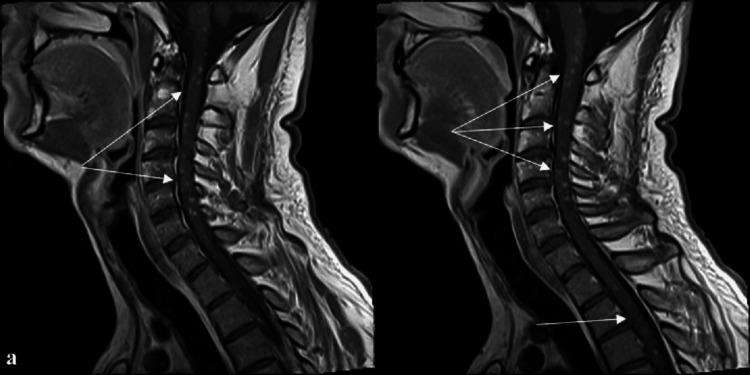
Sagittal T1 MRI of the spine showing multiple round hyperintensities in the cervical and upper thoracic spinal cord MRI: magnetic resonance imaging

**Figure 2:**
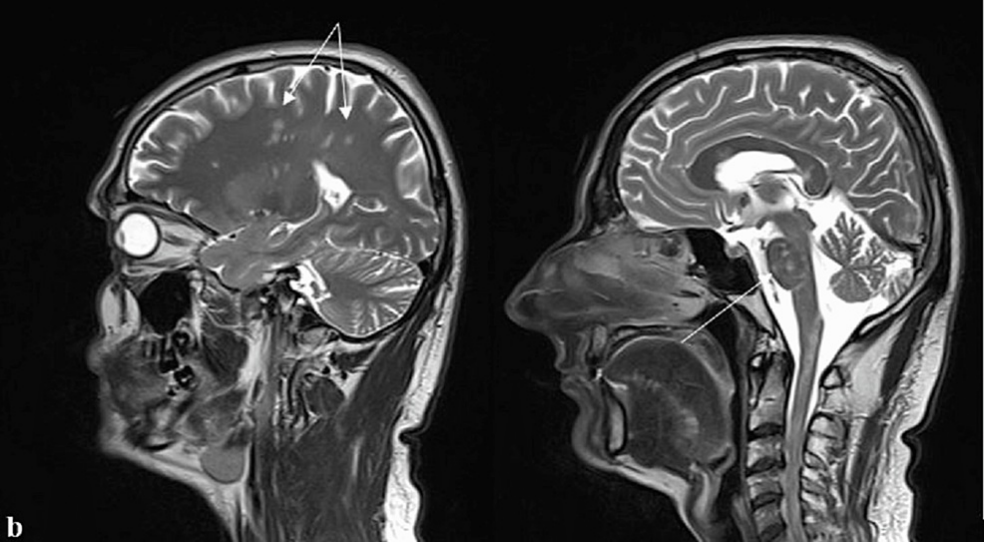
Sagittal T2 MRI of the brain showing multiple hyperintense lesions in the pons and subcortical white matter MRI: magnetic resonance imaging

**Figure 3:**
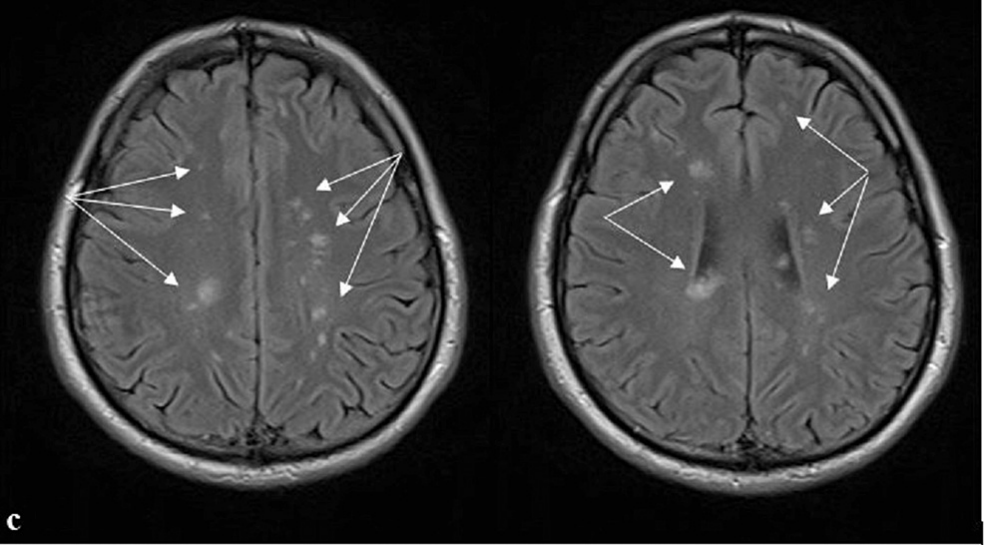
Axial FLAIR MRI of the brain showing multiple hyperintense lesions in the periventricular and juxtacortical white matter FLAIR MRI: fluid-attenuated inversion recovery magnetic resonance imaging

Electrocardiogram, chest X-ray, abdominal and pelvic ultrasound, electromyography (EMG), and nerve conduction studies revealed no abnormalities. Repeated MRI of the brain and spinal cord revealed multiple demyelinating lesions without contrast enhancement in the periventricular and juxtacortical white matter, pons, and cervical and superior thoracic spinal cord (Figures 1, 2, 3). These lesions were superimposed with those from the first MRI. After the treatment with intravenous methylprednisolone (500 mg daily for five days) and B-complex vitamins for five days, the patient had shown a slight improvement in symptoms and discharged home.

In February 2020, he presented to our clinic for re-evaluation, with persistent distal paresthesia of the lower limbs, mild muscle pain in the thighs, and MRC grade 4+/5 spastic paraparesis. During the hospitalization, the blood count, biochemistry, and urine analysis were all with no pathological changes. Electrocardiogram, repeated electroneuromyography, and Doppler ultrasound of the arteries of the lower extremities did not reveal any pathological findings. The electroencephalographic study had revealed a reactive, symmetric, posterior dominant alpha rhythm of 9 Hz, without asymmetry or epileptiform discharges. The ophthalmological assessment had revealed no signs of cataract or fundus abnormalities. Moreover, the patient had been evaluated by a psychiatrist who highlighted a mild cognitive impairment with a mini mental state examination (MMSE) of 25/30 points and mild depression (Beck Depression Inventory - 16 points).

The patient was discharged from the hospital on antidepressant therapy and 64 mg of oral methylprednisolone for two weeks with weekly gradually tapering until cessation. At the regular medical evaluations, the patient showed improvement in muscle pain and paresthesia, but with persistence of the motor deficit in the lower limbs.

Given that the other etiologies of demyelinating diseases of the CNS were excluded, we have interpreted this case and all accompanying pathological findings as consequences of the electrical injury.

## Discussion

There are several case reports and a few comparative groups, most of them being retrospective comprehending neurological complications of electrocution. Here, we are presenting a rare case of delayed CNS demyelinating disease in a patient electrocuted with a low-voltage electrical source.

Electrical injuries occur as a result of direct contact with an electrical source or with a material that serves as a conductor. Although most electrical injuries occur in the low-voltage range, people being exposed to it much more frequently (100-240 V: standard household current), the CNS injuries are more often described with high-voltage current, given that low electrical field strength is seldom enough to produce significant neurological injuries. To the best of our knowledge, this is the first reported case of a delayed-onset demyelinating disease of the brain and spinal cord after low-voltage AC electrical injury [1,10].

Warenits et al., in their analysis of low-voltage electrical injuries, have found that affected patients often present with mild or no symptoms and 73% of them are being discharged within 24 hours from admission. The most important electrocution-related findings were entry marks, neurological symptoms, burns, pain, and cardiac symptoms. Likewise, our patient had suffered only a minor hand burn and had been discharged within the first 24 hours [11].

When an electrical current enters the human body, it travels from the entry point to the grounding point. One epidemiological survey of low-voltage electrocuted patients in China has revealed that the upper extremity is the most commonly involved contact site. However, head-to-anywhere pathway is the most frequently involved one in the development of CNS complications [3,12].

A broad spectrum of CNS and PNS disorders caused by electrocution have been described in the literature. Mononeuropathy is the most commonly reported complication of the PNS involvement, followed by polyneuropathy, polyradiculopathy, carpal tunnel syndrome, and cranial nerve palsies (especially III, IV, VI, VII, VIII, IX, and X). Vertigo, migraine, cerebellar syndromes, epileptic seizures, stroke, encephalopathy, diffuse white matter changes, movement disorders, transverse myelopathy, and amyotrophic lateral sclerosis are some of the complications related to CNS involvement [6]. Neuronal histopathological findings usually revealed in these patients are focal petechial hemorrhages, expansion of the perivascular space, peripheral neuronal fragmentation, damage to the vasa nervorum, and ballooning of the myelin sheath. The electrical injury is not uncommonly associated with a potential heart damage as well. Some authors have described hypercontracted-hyperdistended myocytes, rupture of myocardial fibers, and contraction band necrosis [13].

The neurological complications may occur immediately or later on, and symptoms may be permanent or reversible. A large register-based, matched cohort study has pointed out that the majority of neurological complications occurred within the first six months after the electrocution, but delayed neurological symptoms are also being reported in some cases [4].

In a Canadian multicenter study of previously hospitalized electrocuted patients, 9% had reported muscle weakness, 6% tingling in the extremities, and 9% numbness of the limbs at one year follow-up [14].

The proposed mechanisms of these delayed consequences are vascular spasm, neuronal ischemia, and chronic inflammation of the vascular endothelium [6]. Electroporation, electroconformational denaturation of proteins and DNA, and neuroexcitotoxicity due to glutamatergic hyperstimulation also play an important role in vascular and demyelinating changes in the CNS [7].

General epidemiological reports regarding age, sex, type of electrical current, circumstances, and place of electrocution correspond to the data presented in our case. Making this case special is the fact that the patient had been electrocuted with low-voltage AC, which is exceptionally involved in the development of CNS complications. This type of electrical current is potentially more dangerous and could cause wider extensive tissues damage than high-frequency current, as AC tends to cause local tetanic muscle spasms, which stick the patient to the electrical source and affect the cardiovascular and respiratory systems. Although AC causes significant damage to the heart and lungs, our patient was spared from these complications. Although the neurological complications occur most frequently with the head-to-anywhere pathway, in our case, the entry point was the upper extremity, which is also the most commonly involved site of contact in electrical injuries.

While in most cases, symptoms appear immediately after the electrocution or within the first six months, in our case, they came into sight one year after the electrical injury. Although we found separate cases of diffuse white matter changes in the brain and also transverse myelopathies associated with the electrical injuries in the literature, we did not find brain and spinal cord involvement at the same time. Thus, more patients with CNS complications after low-voltage AC electrocution studied are required to further elucidate the exact mechanisms and pathophysiology of electrical injuries.

## Conclusions

According to the available data, most of the neurological complications related to electrical injuries occur immediately after exposure to high-voltage electrical current. Even so, CNS involvement is described in electrocuted patients regardless of the type of current, voltage, and time from exposure. However, even if the incidence of neurological complications is lower with low-voltage AC, these should not be neglected as they can still occur in a minority of patients. To the best of our knowledge, we reported a first case of a delayed demyelinating disease of the CNS following low-voltage AC electrical injury. In order not to delay the specific treatment, an exhaustive differential diagnosis workup was performed, excluding the most common etiologies of the demyelinating diseases of the CNS. In conclusion, these patients should be carefully monitored not only in the acute phase but also over a longer period, seeing that, as reported in this case, neurological complications may occur later after electrocution.
